# How do exhausted parents experience their interactions with their children? A qualitative and participative study

**DOI:** 10.3389/fpubh.2024.1340748

**Published:** 2024-05-01

**Authors:** Anne-Catherine Dubois, Margaux Roberti-Lintermans, Zoé Mallien, Aurore François, Magali Lahaye, Jan De Mol, Isabelle Aujoulat

**Affiliations:** ^1^Institute of Health and Society, Université Catholique de Louvain, Brussels, Belgium; ^2^Institute for the Analysis of Change in History and Contemporary Societies, Université Catholique de Louvain, Louvain-la-Neuve, Belgium; ^3^Psychological Sciences Research Institute, Université Catholique de Louvain, Louvain-la-Neuve, Belgium

**Keywords:** parental burnout, parents, children, interactions, loss of control, suffering

## Abstract

**Introduction:**

Parental burnout, known as a state of physical and psychological exhaustion, results in an imbalance between the parent’s perceived stressors in relation to parenting, and the resources available to the parent to cope with such stressors. The causes and consequences of parental burnout for the parents themselves have been studied from the parents’ point of view, but the perception of parents regarding the impact of parental burnout on the parent-child relationship has not yet been documented.

**Methods:**

We conducted a qualitative study through semi-structured interviews with exhausted parents (n=21). We aimed to better understand their general interactions with their children, as well as the way they communicate with them about their state of exhaustion, knowing that dealing with parental suffering can have a long-term impact on the child.

**Results:**

Our results reveal that exhausted parents experience a widespread loss of control in all areas of their lives, particularly in their interaction with their children, which generates feelings of guilt and shame. Communicating their experience to their children can create various difficulties for both parents and children. This may complicate the process of seeking help and reinforce the feeling of isolation.

**Discussion:**

An emerging result from our analysis leads us to identify a need for the parents to be heard and validated in their suffering who took part in this research.

## 1 Introduction

Parental burnout (PBO) defined as a state of physical and psychological exhaustion of the parent, has been described by psychologists and researchers as a syndrome related to stress experienced by parents in parenting, resulting in an imbalance between parental stressors and the resources available to the parent to cope (1).

The emergence of parental burnout is part of a historical evolution in the concept of parenthood (2) and is related to the place given to children in our societies (3). PBO is known to impact parents’ physical health (e.g., sleep disorders, physiological complaints), mental health (e.g., addictive behaviors, suicidal ideation), and well-being (e.g., reduced psychological well-being, low sense of life satisfaction) (4, 5). A combination of socio-economic, family, and/or personal characteristics is associated with PBO (6). At the socio-economic level studies have highlighted, among other things, the stress felt by parents in balancing their family and professional lives (7), and low income (8). At the individual level, authors have shown that a tendency toward self and socially prescribed perfectionism (9) and low emotional intelligence scores (10) are associated with a higher risk of PBO. At the family level, parenting a child with specific needs (chronic illness, disability, learning disabilities, behavioral disorders, emotional difficulties) increases the risk of developing PBO (4, 11).

Regarding the quality of social and family relationships, studies have shown that parental exhaustion affects romantic relationships, particularly by causing partner estrangement or increasing tensions and conflicts (4, 12), creates role confusion, and discourages asking for or receiving social support from family and friends (5, 13).

Much less is known regarding the interactions of exhausted parents with their children. The results of two separate studies suggest that parents may externalize their exhaustion through behaviors that could ultimately lead to neglect, as well as verbal or physical abuse (14, 15). Although these findings highlight the potentially adverse consequences of PBO on children and parent–child interactions within the family, this issue remains largely under-investigated in the literature (16). Moreover, while many quantitative studies have examined the various facets of PBO, few research studies have questioned parents in depth, particularly about their interactions with their children and how they communicate their exhaustion to them. Therefore, a better understanding of the impact of PBO on the child and the parent–child relationship remains a major challenge for societies that place children and their well-being at the heart of their concerns and public policies. Given the knowledge gap in the literature, we wanted to understand what parents themselves say about parental burnout and how they talk about their children’s experiences when they are faced with parental exhaustion.

## 2 Materials and methods

The study of parents’ experience of their interactions with their children in the context of PBO is a complex and delicate issue. To date, the parents’ perceptions of the consequences on their interactions with their children remain yet an underinvestigated issue. Inviting burnt-out parents to talk about their experiences, feelings and perceptions of their parenting and family dynamics requires special care, so as not to further undermine their perceived parental competence. That is why we opted for qualitative methods, as these are particularly suited to explore sensitive and underinvestigated issues related to human experience (17), without imposing predefined theoretical categories on them, as is the case with survey questionnaires.

### 2.1 Recruitment methods

Potential participants were recruited through a network of professionals who disseminated the information through written materials, posts on social media, or personal contact with the families. A total of 56 professionals were personally met by the first author and agreed to act as facilitators in the recruitment phase. The professionals gave the information to the parents, who contacted us by e-mail or telephone to set up an appointment for the interview, once they had indicated their interest in the research and their agreement to participate.

### 2.2 Sample characteristics

To be included in our study, the participants had to be the parents of one or several children aged less than 18, in health (HC) or with a chronic illness or disability (CC), be aware of being exhausted in their parental role, be willing to share their experience with us, and be fluent in French.

Following an initial contact by phone or e-mail with 28 parents, 21 parents (13 individual parents and 4 parental dyads) eventually agreed to participate. The drop-out of the 7 remaining parents was due to a lack of time (*n* = 4) or refusal by the partner (*n* = 1). A further 2 parents did not follow up after the first contact, despite the first author’s efforts to keep in contact. Among the 21 parents, 11 parents had HC, 7 parents had one (*n* = 6) or two (*n* = 1) children with CC and 3 parents reported learning difficulties or behavioral disorders (LBD) in one (*n* = 2) or two (*n* = 1) of their children. The characteristics of the participants and their families are presented in Table 1.

**Table 1:** Summary of participants’ characteristics at time of interviews (*n* = 21).

Parents	
Variables	*N* (%)
Sex	
Female	17 (81%)
Male	4 (19%)
Age	
30–39	10 (48%)
40–49	10 (48%)
50–59	1 (4%)
Education	
University studies	10 (48%)
Non-univeristy studies	11 (52%)
Employement status	
Employed full-time	6 (29%)
Employed part-time	8 (38%)
Unemployed	2 (9%)
Invalidity due to PBO	5 (24%)
Current PBA score	
Burnout	8 (38%)
Probably at risk	5 (24%)
Moderate risk	3 (14%)
Low risk	4 (19%)
No parental burnout	1 (5%)
Psychological support	
Present	15 (72%)
Past	3 (14%)
none	3 (14%)

Families	
Variables	*N* (%)
Type of family	
Traditional	10 (59%)
Stepfamily	2 (12%)
Single parent	5 (29%)
Self-assessed standard of living	
Very at ease	6 (35%)
Average	7 (41%)
With financial difficulties	4 (24%)

Children			
Groups	Number of families concerned (*n* = 17)	Number of children (*n* = 38)	Age [mean, (min-max)]
Families with healthy children	8 (47%)	27 (71%)	10y [2–19]
Families with children with chronic conditions	6 (35%)	7 (18%)	9y [5–14]
Families with children with behavioral or learning difficulties	3 (18%)	4 (11%)	10y [8–12]

In addition to general sociodemographic characteristics, the results of the Parent Burnout Assessment (PBA) (18) are also presented in Table 1. The PBA was not used as a recruitment tool to validate the parent’s inclusion in the study, but to characterize our sample. This means that parents with a low PBA score who wished to participate in the research were nevertheless included in our study. From an ethical point of view, we felt that by doing so, we were enabling parents to be recognized and heard in their suffering.

### 2.3 Data collection

We conducted 17 in-depth interviews, both with individual burnt-out parents (*n* = 13) and with parental dyads, both in PBO (*n* = 4 parents), followed by a focus group to discuss our results with the concerned parents.

#### 2.3.1 Interviews

An interview guide was developed around two main open questions to ask participants about how they experienced their state of exhaustion, and how they viewed their interactions with their children as a result of this. The in-depth interviews were conducted either virtually on Teams (*n* = 16) or at the researcher’s office (*n* = 1), according to the participants’ preferences. The mean duration of the interviews was 69 min (min. 36 – max. 125).

#### 2.3.2 Focus group

A focus group discussion (FGD) took place to discuss with the participants the results and hypotheses generated by the analysis of the collected data. This FGD took place after an average number of 14 months (min. 10 months – max. 17 months) following the in-depth interview. Out of the 21 parents who had participated in an interview, 10 parents representing 8 families, agreed to participate in the FGD. Of the 9 remaining families, six participants were unavailable at the time of the focus group, one participant was not interested, one participant did not respond to the invitation, and one participant canceled at the last minute due to unforeseen circumstances.

The FGD of parents gathered 6 individual mothers and two parental dyads, representing 8 families, of which two had a child with LBD and two had a child with a CC. The FGD lasted 1 h and 38 min.

After setting up a safety framework, a PowerPoint presentation was used to expose the research findings to the parents. We used different tools designed by our research team, like a visual scale, to make exchanges between parents more concrete and active. Participants were invited to discuss and validate our results.

### 2.4 Analysis

All interviews were transcribed verbatim. The transcripts were analyzed according to the principles of phenomenological interpretive analysis (19). The first and last authors analyzed the two first interviews independently and agreed on a starting list of thematic categories to describe how the participants experience being exhausted in relation to their children and how they view their interactions with their children. As a result of this first step of the analysis, a slight evolution of the interview guide was agreed to elicit more precise responses in relation to the parents’ interactions with their children, as well as how they would describe and experience their children’s behaviors. Further, all interviews were read and discussed between the first and second author, and during co-authors’ meetings, to discuss emerging results. These discussions were intended to enrich the understanding of what had been conveyed by the participants, not to reach a consensus (20). An additional way of validating the results was implemented during the focus group through feedback and validation of the results by the participants. In addition to researcher and participant triangulation in the process of analysis, the first author also regularly met with the sixth author, a clinical psychologist, and lecturer in family psychology. This was to support reflexivity and minimize the risk of subjectivity bias, discussing emotional and possible transference issues arising during the interviews.

Following these multiple reviews and discussions, four meaningful categories of analysis were created: (i) losing control in interactions and communication with their children, (ii) encountering difficulties in communicating with their children about their experience of parental burnout, (iii) trying to make sense of their current experiences by looking at their past experiences as children, and (iv) a need to be heard and validated in their suffering: an emerging result.

### 2.5 Ethical considerations

Our research protocol was approved by the ethics committee of our university (ref. 2021/24JUI/286). An informed consent form was signed by the parents. In order to guarantee their psychological safety and not reinforce their guilt, we had the possibility of directing parents to professionals trained in supporting parental burnout, if they wished.

## 3 Results

Our results are presented according to the four themes listed above, namely (i) losing control in interactions and communication with their children, (ii) encountering difficulties in communicating with their children about their experience of parental burnout, (iii) trying to make sense of their current experiences by looking at their past experiences as children, and (iv) need to be heard and validated in their suffering: an emerging result. The results are illustrated by quotes from the parents during the interviews or the FGD who are identified as parents of healthy children (HC), parents of children with chronic conditions (CC), or parents of children with learning or behavioral difficulties (LBD).

### 3.1 Losing control in interactions and communication with their children

First, it is to be acknowledged that the participants reported experiencing a widespread loss of control that would impact their social roles in their personal, professional, and family lives: “*It is exhausting! When you slip up on one side, it affects your other identities”* (Parent of a child with LBD, FGD) Yet, as the difficulties described by exhausted parents in their personal and social lives have already been documented in the literature, the results presented hereafter mainly focus on the burden explicitly related to the interactions between parents and their children, the loss of control in these interactions, and the way parents perceive the consequences of this loss of control on their children, particularly when describing their reactions.

Some parents reported that the emotions and tensions experienced in their interactions with their children would sometimes lead to losing patience, shouting, crying, as well as verbal or physical loss of control manifested through hurtful words or inappropriate gestures (e.g., grabbing the child, hitting the child, throwing objects across the room). “*It happened to me once that I hit one of my children and after I realized what I had just done, it followed me for a long time, to see how far I had gone to get to that point*” (Parent of a child with CC, interview). Such losses of control were described by the participants as a major symptom of their exhaustion, which would act as a real alarm bell: “*There is nothing worse*” (Parent of a HC, FGD). Some participants reported awareness of the impact of their attitudes on their child’s behavior: “*She had to make up for my unavailability for such a long time*” (Parent of a HC, interview). In connection with their loss of control, some participants noticed that their children would feel alarmed or be prone to panic as soon as they would raise their voices (Parent of a HC, FGD). Moreover, even when everything was fine, some children would ask back “*Are you okay, Mom?*” or “*Is there anything I can do to help?*” (Parent of a child with LBD, interview). Some participants mentioned their children’s emotional reaction when they lose control: “*She also went through a phase of feeling angry, so angry… this went on for several months*” (Parent of a HC, interview). “*For our second child, if we are not doing well, my husband or I, it is an explosion (...) He takes on all the emotions (...) In the beginning, we did not see that. We just saw a child who was in crisis in addition to what we were going through*” (Parent of a HC, interview). Moreover, some participants reported that their children would sometimes reproduce their parents’ behaviors in losing control when relating to their siblings (e.g., arguing, shouting) “*It is confrontational and very complicated*” (Parent of a HC, FGD).

By contrast, other participants identified the development of a protective attitude among the siblings, in reaction to the parent’s loss of control: “*They both love each other, they have a great bond*” (Parent of a child with CC, interview) or a protective attitude of the children toward their parents. “*My daughter tends to protect me when I’m exhausted*” (Parent of a child with CC, interview). For some participants, the protection given by their child could lead to “*a savior attitude on the part of the eldest one*” (Parent of a child with LBD, FGD).

Regarding their interactions with their children, some participants feared that their loss of control might leave traces of trauma in their children’s adult life. This in turn would further increase their guilt and helplessness: “*I feel that for her [daughter] this period has been traumatic*” (Parent of a HC, interview) or “*When she expresses doubts about herself, I ask myself: ‘Am I responsible for this as a parent?’*” (Parent of a HC, FGD). The participants also listed the responsibilities that would rest on their children’s shoulders when exposed to parental exhaustion: “*I ask a lot of him in the end, I realize that in order to prevent me from going crazy, I ask a lot of him*” (Parent of a child with LBD, interview) or the expectations they have of their child: “*I had to get them to participate in house chores*” (Parent of a HC, interview).

### 3.2 Encountering difficulties in communicating with their children about their experience of parental burnout

Parents find it difficult to communicate about their experience with their children. The feelings of shame and guilt that were reported by the participants in relation to their experiences of losing control were further described as making their experience of exhaustion barely communicable to their children: “*I feel guilty almost all the time*” (Parent of a HC, interview). The participants described how they would come to doubt themselves, and to be afraid of failing in their parental role, to the point of feeling that they are no longer up to the task. “*I have always had doubts about what I was doing, from the very beginning of parenthood*” (Parent of a HC, interview). Some participants even said they no longer recognized themselves, that they felt they had become strangers: “*I had become someone I was not, a person who was unknown to me …*” (Parent of a HC, interview). Moreover, the participants also talked about sadness, crying, and a sense of failure that would undermine their self-esteem: “*You have no understanding of yourself, you think you suck*” (Parent of a child with behavioral or learning difficulties, interview). All this, they said, would contribute to making their experience uncommunicable, in particular to their children.

To talk about their experience of PBO, to find words to explain this psychological state to their children, and to name their emotions in such a situation is no easy task for burnt-out parents. Some participants reported that they would not reveal in front of their children that they were exhausted. “*Talking about it, no, we do not talk about it*” (Parent of a HC, interview). Other participants sometimes resort, without always being aware of it, to emotional communication centered on their own experiences or feelings *“‘I cannot take it anymore’, I would often say to my children, before staying on my own for a little while* (Parent of a HC, FGD). They talked about their difficulties in putting into words their experience of losing control, in explaining to their children that they are not responsible: “*I still have trouble reassuring him with this (...) It is not a life, well, it is not nice for him (...) My son has always tested the limits because he never felt safe*” (Parent of a child with LBD, interview). They spoke of their difficulties in recreating a bond with their child following an outburst: “*Telling your child that you are sorry is not enough*” (Parent of a child with LBD, FGD). When they lose control while interacting with their children, either physically or verbally with emotionally loaded communication, participants tended to think that “*the child does not understand that he/she is not the cause of our anger*” (Parent of a HC, FGD).

Some participants reported communicating their feelings to their children, acknowledging that this might be too much for their children to some extent: “*Sharing my emotions with my children has become more and more important over time. But I think I feel the need to share more than my children need to hear what I’m going through*” (Parent of a child with LBD, FGD).

Some participants reported not having any filter while sharing their own emotional experiences with one or several of their children. They tend to explain everything to their children, often inconsistently between children, as the eldest most of the time would receive more explanation than the younger and sometimes even be designated by the exhausted parent as his/her confidant: “*I really do talk to them without secrecy (...) I think I have really confided in them a lot more, especially with my eldest child*” (Parent of a child with LBD, interview). It should be noted that the quality of the communication may vary, as this participant indicated: “*Sometimes, I manage to say it, to verbalize it. Perhaps more with my little one (who is in good health) than with my older one (who has a CC). And sometimes I cannot, and I tell them I need a moment to myself, and I close the door. And sometimes I do not even tell them, and things get out of hand*” (Parent of a child with CC, interview).

Tuning in to their children’s own experiences was also described as difficult by some: “*When he expresses himself, I cannot listen*” (Parent of a child with LBD, FGD). Conversely, some participants reported that they would give their children tools to express their emotions but fail to express their own feelings: “*Every child has his own ‘emotion cards’ with the seven basic emotions, and every evening we take out the cards and they express their emotions*” (Parent of a child with LBD, interview).

### 3.3 Trying to make sense of their current experiences by looking at their past experiences as children

Reflecting on their interactions with and responsibilities toward their children, most participants recalled certain emotional experiences from their own childhood, linked either to adverse events or to their own parents’ parenting style. When they realize the impact of their loss of control on their children, they cannot help but think of what they experienced or felt during their childhood in the face of similar or different events that left their mark. It seems that family history has an impact on the way parents interact with their children. Some participants reported that they had experienced a parenting style that they described as being demanding, rigid, authoritarian, and perfectionist: “*With my parents, it was ‘The more perfect you are, the more we will love you*’“(Parent of a HC, interview). Some participants linked these childhood experiences to difficulties in expressing their own emotions. They reported to have felt pressure and anxiety during their childhood, and often a lack of space to express their own emotions, experiences, and feelings. “*We had no space for emotion*” (Parent of a HC, interview) or “*What you also missed was being able to put things into words. You did not talk about your feelings in your family*” (Parent of a child with CC, interview). These participants reported that their own parenting styles tended to be shaped in part by that of their parents, but not exclusively: “*It is not as if I would dismiss it all; rather I tend to add to what I received*” (Parent of a HC, interview) or “I *know that I make high demands on myself and that comes from my education*” (Parent of a child with CC, interview).

Other participants, on the contrary, described the education they had received as chaotic, and neglectful, with a lack of structure and limits. The recalled lack of presence and structure was acknowledged as a source of pressure for their own parenting by those who had experienced it: “*Clearly, my parents were not present at all. And so I do not want to put my son through that*” (Parent of a child with LBD, interview).

A few participants reported having experienced traumas during their childhood, related to the mental health problems of their parents. They think that this experience contributes to their parental burnout and influences their loss of control, similar to those they observed in their parents during their childhood: “*I started carrying my mom on my shoulders at the age of 10. My mom had psychiatric problems, physical problems as well*” (Parent of a child with CC, interview). “*My dad had major depressive episodes when I was young, which is probably why I wasn’t very close to him. But I never knew about it, and it was only as an adult that I really understood some of it*” (Parent of a child with LBD, interview); “*My father died 15 years ago. He committed suicide*” (Parent of a HC, interview). On several occasions, it was not until the participants had become parents themselves that they became aware of the impact of family trauma on their own lives: “I *experienced a lot of traumas, however, it took some time before I became aware of it. (...) My therapist once said to me: ‘You’re in survival mode*’“(Parent of a child with LBD, interview). Sometimes family secrets were discovered or understood later in adolescence or adulthood: “*There are really very difficult things that have happened in the family and that had never been discussed, a lot of unspoken things and even family traumas that are passed on from one generation to the next one without being understood because they remain unsaid”* (Parent of a HC, interview).

Thus, the parents with a difficult personal and family history in our sample were inclined to try and fix what had been broken in their own childhood by putting a lot of pressure on themselves to do better than their parents. “*I try to correct what my parents did not do right*” (Parent of a HC, interview).

Some participants tended to view their own parents as superheroes whose level of perfection they could never reach: “*I really did not want to give the kids the feeling I had, that parents are everyday superheroes and that they never fail*” (Parent of a HC, interview).

Finally, childhood experiences also influence interactions within the couple, sometimes creating additional communication difficulties with their children on top of those already experienced. For example, if the two partners had contrasting experiences of parenting styles and family models in their own childhood, this could generate additional stress and even conflicting communication: “S*he comes from a very loving family, it was the opposite in my family. As a result, our way of seeing things often oppose. Something that does not seem very serious to me will be serious to her and* vice versa” (Parent of a child with LBD, FGD). Moreover, a traumatic event experienced during childhood, such as the absence or loss of a parent, calls into question the place given to the spouse in the parental couple, as one mother who was raised without a father explains: “*Oh yes, there’s a father here! What place should I give him or not give him? While it suits me well to manage my own things, I tend to blame him afterward for it: ‘But you did not do anything!’ If I have taken up all the space, it is a bit difficult to ask my spouse to be there to*o” (Parent of a HC, FGD).

### 3.4 Seeking to be heard and validated in their experience of parental burnout: an emerging hypothesis of our results

The intensity of the feelings of shame and guilt that the participants associated with their experience of losing control, which would lead to making their experience incommunicable to their children and in turn add feelings of isolation to the burden of feeling exhausted, led us to question whether the experience of burnout might be perceived as something that should not be experienced and may not be expressed. This hypothesis was further discussed and validated during the focus group: “*I do not feel legitimate because I think I’m the problem’*“(Parent of a HC, FGD).

During the interviews, the parents expressed their need and difficulties in finding circumstances that in their eyes would justify their exhaustion or give them the right to be exhausted. According to the participants, parents have no right to be exhausted in their parental role: “*To be a good parent, I told myself I had to do everything: And I have to go to the dump, and I have to do the laundry, and I have to do the shopping, and I have to cook, and this and that...*.” (Parent of a HC, interview).

They tended to judge themselves, to compare themselves with other parents who were doing better while experiencing similar difficulties. These different elements were confirmed during the FGD, where parents expressed that they believed they were the problem at the heart of the situation while struggling to identify valid reasons for exhaustion in the eyes of society.

Our results suggest that participants who were able to point out difficulties in their lives that would justify their exhaustion were able to judge themselves kindlier than those who could not identify any mitigating circumstances. To be able to identify an external cause that explains exhaustion seems to reduce the parent’s feelings of shame, guilt, and self-blame. In our sample, parents of a child with CC, parents of twins, and single parents reported a lower sense of guilt and less influence from perceived social pressure: “*Learning that my wife was pregnant with twins, that the third one had turned into three AND four, we had not thought of that. Very clearly, the feeling of fatigue is there since they are four and I never had this feeling before, never. In terms of logistics, it is clear that everything is multiplied by four. So it is exhausting*” (Parent of a HC, interview). Yet, they would still tend to compare their own situation with that of other parents whom they would consider having more reasons (and rights) to be exhausted than themselves. “*Knowing that my situation is complex, and help is not always available, I feel relatively legitimate. I tend to compare myself to other families who are going through more difficult things, which is why I do not feel entirely legitimate*” (Parent of a child with CC, FGD).

The parents who had a child with a CC reported feeling more pressure from their child’s chronic condition than social pressure linked to their parenthood.

Some parents reported to have needed a medical order to accept a prescribed work break and feeling less shamed to stop work: “*The fact that a doctor stops us makes the situation legitimate and shows us that we are not superheroes*” (Parent of a child with LBD, FGD). The presence or absence of a partner was another mitigating factor reported by the single mothers in our sample who were raising their children alone that would justify an increased risk of exhaustion and make it more acceptable: “*I am a single mum (...) It was one of the elements that generated burnout* » (Parent of a HC, interview).

The parents’ interviews, illustrated by the various verbatims above, show that exhausted parents do not give themselves the right to be exhausted, or to talk about it, which reinforces their sense of isolation. Based on our results we formulated the hypothesis of a need for parents to be heard and validated in their suffering, which was validated by the parents in the focus group. Parents expressed that they need to feel they have right to be exhausted. They also validated their difficulties in talking to their children about their experiences and emotions. At the end of this study, we believe that a greater sense of validation of their suffering could have a positive impact on feelings of isolation and loss of control, and could perhaps change the way parents communicate with their children.

## 4 Discussion

Our results highlight that exhausted parents experience a widespread loss of control. This loss of control, which occurs in different spheres of their lives and especially in their interactions with their children, gives rise to feelings of shame and guilt, among others, which impact further interactions with their children. Such feelings may be influenced by emotional experiences from their own childhood. When parents lose control of their interactions with their children and feel ashamed and guilty, they tend to flee or isolate themselves. Communicating their experiences to their children and talking to them about their emotions, while listening to their children’s own experiences, can create various difficulties for both parents and children. This may complicate the process of seeking help and reinforce the feeling of isolation. An emerging result of our analysis that was validated by the participants during the focus group led us to hypothesize that burnt-out parents may experience difficulties in being heard and validated in their suffering, which is likely to impact their loss of control, their sense of isolation, and maybe the way they communicate about their experience to their children. All this can delay their request for help for themselves and their children.

### 4.1 The repercussions of loss of control

Our results reveal a loss of control in the exhausted parents’ interactions with their children. While a risk of violence or neglect has been reported in relation to parental burnout (15), our results point to the suffering experienced by exhausted parents as they lose control – or fear to lose control – in their interactions with their children. This echoes the results of another qualitative study, which evidenced that suffering and fear were central to the experience of burnt-out mothers (14). We wish to emphasize the importance of carefully choosing the words to use when addressing parents and society about PBO and when discussing possible consequences for their children. Inviting parents to reflect and talk about possible losses of control and the suffering associated with it might be a way to open the dialog about parents’ suffering, in an empathetic and non-judgmental posture less likely to stigmatize the parents, thus contributing to support the parents’ self-awareness and a need for help.

It is also important to remember that witnessing parental loss of control as a child can be traumatic. The experience of adverse events in childhood can lead to trauma and impact a child’s security and attachment, resulting in a decreased ability to regulate emotions in adulthood and difficulties in relating to others (21–23). A traumatic childhood event, regardless of its intensity, if left unresolved, can be reactivated during adulthood or parenthood. We have observed a tendency among suffering parents to re-interrogate their own childhood experiences and their own suffering. This highlights the importance of giving children a voice, to enable them to express and understand what they are going through when their parents are exhausted. Moreover, the participants in our study acknowledged facing difficulties in expressing or regulating their emotions, sometimes specifying that there had been no room for emotions in their childhood. Emotional competence has been identified as a protective factor for PBO (4, 24). These authors emphasize the crucial role of emotions for the survival of the individual, as emotions inform us of our needs and invite us to respond to them. They further point to the need to become aware of one’s own emotions and to communicate them in an appropriate manner to avoid becoming overwhelmed by them and being able to identify other people’s emotions, dealing with them appropriately. This is another essential competency for the parent–child relationship, which needs to be promoted to help parents come to terms with their state of exhaustion and the consequences of it.

### 4.2 A need for parents to be heard and validated in their suffering: an emerging result

As already mentioned, our study generated the hypothesis that burnt-out parents may experience a need to be heard and validated in their suffering that is likely to impact their ability to communicate about their experience and seek help for themselves or their children in a timely manner. To the best of our knowledge, scientific literature on burnout sufferers’ sense of validation of their lived experience is rather scarce. The legitimacy of professional burnout has been studied by Friberg in Sweden in 2009. Friberg identifies two processes by which the concept of burnout moved to a psychiatric diagnosis, making this state of exhaustion more acceptable: (i) the scientific investigations that lead to the diagnosis, and (ii) the responses made by political decision-makers to improve the preventive and curative management of this diagnosis (25). From a public health perspective, to be perceived as legitimate, the suffering caused by a disease must be recognized both explicitly and socially and must have been scientifically explained (26). This brings us back to the social construction of illness as described by Monaghan & Gabe (27) as “*the way in which people experience illness, recognize and interpret symptoms, interact with their* var*ious networks, by coping with and accommodating these symptoms*.”

In this respect, we could consider that the multiplicity of symptoms and effects associated with PBO, as Kirouac (28) has highlighted for burnout at work, may lead to a rather vague definition and symptomatology, presenting general realities that are not exclusive to PBO (e.g., relationship problems, tensions, withdrawal, etc.). This ‘vagueness’ makes it difficult to distinguish between those who suffer from it and those who do not and leads to skepticism about its existence as a syndrome, and even suspicion of those who claim to suffer from it (28). In the same vein, it should be remembered that the participants in our research who felt the greatest suffering were those for whom this ‘vagueness’ was all the greater because they could not clearly identify any causes, such as a child’s illness for instance. This corroborates the rationale behind our decision to include parents who were not diagnosed according to the PBA test, but who nevertheless expressed distress in their role as parents. The discrepancy between the scores obtained with the PBA scale and the experience reported by the participants demonstrates some restrictions in a measurement tool used to diagnose PBO. Kirouac (28), speaking of the psychometric tools used to diagnose professional burnout, notes that they fail to identify the psychological needs of the individual and are more a reflection of the expectations and constraints that characterize the experience of contemporary work. When it comes to family dynamics, standardized measurement tools may be insufficient because the situation being studied is too complex (28). Position on professional burnout seems therefore fully transferable to PBO: the essential thing is to take it seriously as a social phenomenon that reveals contemporary attitudes to parenting – namely that some parents associate suffering with it.

As for professional burnout, and in addition to the factors related to one’s personal history, as reported in our results section, the parents’ sense of being heard and validated in their experience of exhaustion may be influenced by perceived social pressure and the normative demands conveyed by society (29). Indeed, the many expectations perceived by parents in their environment may constitute a source of considerable pressure, even if they apply to them in very different ways (30). The tendency to make parents responsible for the future of their children is not a new phenomenon in the history of industrialized societies. To take the example of Belgium, where this study was carried out, it should be remembered that at the turn of the 19^th^ and 20^th^ centuries, mothers were heavily involved in public policies aimed at reducing infant mortality (31). In the interests of social regulation, the state also took an interest in families considered dysfunctional, and developed measures ranging from support to control, and from accompaniment to punishment (32). Knowledge about children’s development and well-being was built up and widely disseminated, while the family was maintained as the privileged and “natural” place to grow up: the need for a relational (33) and loving family was increasingly asserted throughout the 20^th^ century, in addition to the need to care for and educate children (3). Children, thanks to the availability of contraceptive methods, have become a project in themselves: we no longer “have” children, we “make” them, and their development requires multiple skills, first from mothers (30), then progressively from parents, who are widely considered responsible for the success (or failure) of this development. Even if the political and media discourses tend to support parenthood, the resulting model of the ‘good parent’ leaves little room for mistakes or feelings of failure. Suffering from burnout is not validated since it is still considered a taboo subject, about which there is little communication (34). In addition, they feel obliged to thrive in their other social roles, that of husband or wife, professional, or friend... These different elements make us think that it is important to act by not reducing parental burnout to an individual or family problem but by also considering it as a social fact that deserves attention at the macro, meso and micro levels.

## 5 Strengths and limitations

One of the main strengths of this qualitative study is that it is one of the first to have given voice to parents experiencing burnout, by taking the time to listen to them in a semi-structured interview. Numerous quantitative studies have made it possible to examine multiple facets of parental burnout through questionnaire surveys. Another strength of this research lies in the deployment of data collection and analysis methods where triangulation with researchers, clinical experts, and participants enabled us to develop a collaborative approach that allowed the participants to validate the results. Multidisciplinary interactions and exchanges throughout the research also contributed to enriching our methods and results. Indeed, an important aspect of the qualitative approach is to demonstrate the credibility of the results presented, to reduce the risk of interpretation bias linked to the researchers’ subjectivity (35 36). Subjectivity always comes into play. It is not a bias if the researcher is aware of it (37). To manage subjectivity, the researcher must take a reflexive step back from his/her situation to question the influence he/she is likely to exert on the conclusions he/she will reach (38). A phase of introspection, also known as self-analysis, enables the researcher to become aware of his/her position regarding a phenomenon being studied and to mark a decoupling between him/herself and the phenomenon (39). Another strength of our study lies precisely in the reflexivity that supports the entire research process.

They were sometimes experienced by the participants as an opportunity to put into words their exhaustion or to relive the path taken since they had become aware of their state of PBO.

One limitation of our study concerns our small sample size and limited characteristics, which did not allow us to reach theoretical saturation. Most of the participants were enjoying rather high standards of living (self-reported), as well as having higher levels of education. They were homogeneous from the cultural point of view, with a lack of diversity. Moreover, following the results of our literature review (40), we decided to recruit the parents through our own network of professionals to ensure their psychological safety, to avoid the risk of destabilizing their personal and family balance, as this had already been shaken by the exhaustion they were experiencing. Consequently, we had to deal with parents who were aware of their exhaustion and often already engaged in a process of psychological support. This observation leads us to question how to recruit, and interview in the future, parents who do not recognize themselves as exhausted or who deny it, as well as how to increase the socio-economic and cultural diversity of the sample. The fact that we conducted most of our interviews on Teams to meet the challenges of the COVID-19 pandemic could be perceived as a brake on parent participation and the quality of exchanges. But we found that for exhausted parents, there was something comfortable about not having to travel and staying in their usual environment to talk to us, both in terms of the time constraints parents face, and in terms of emotional security (41). What seems important to us in our context is that the participants feel comfortable with the conditions in which they share their experiences, and that the researcher who interviews and listens to them is trained and supervised in his work. Offering researchers a supervisory space in which to reflect and debrief promotes their well-being (42).

## 6 Perspectives

Further research on a larger sample is needed to better understand what is transversal and specific to the experiences of parental burnout of parents of HC compared to parents of children with CC.

The hypotheses that emerged during this research concerning the need for parents to be heard and validated in their suffering and regarding the way exhausted parents communicate and interact with their children need to be explored from the children’s point of view to better understand the lived experience of children exposed to parental exhaustion. Knowing more about children’s experiences would make it possible to consider support to allow them to express their perceptions and needs in this context of parental burnout.

Also, it seems essential to us to act on the feelings of guilt and shame felt by exhausted parents, by questioning the parents’ personal and family histories, in interviews with psychologists trained in parental burnout, and by allowing them to identify the pressure perceived in their environments to act on them, as part of a health promotion approach. So far, the interventions proposed have been targeted for each parent, on an individual basis. We need to go further than this person-centered approach. To reach this goal, we could draw inspiration from the (48), which defines health promotion as a combination of actions aimed at the individual, through empowerment, participation, and the development of skills, as well as actions in the living environment, involving the political sphere. Furthermore, the methodology of this research could inspire an interesting clinical approach, in particular by allowing spaces for exchanges between exhausted parents, supervised by professionals, as we did in the closing phase of this study.

## 7 Conclusion

Our results suggest that exhausted parents need to be supported to make their experience more recognizable, and therefore more likely to be communicated. Not feeling heard and validated in what they are experiencing could explain parents’ loss of control in interactions with their children, as well as feelings of shame and guilt that may be influenced by emotions experienced during their own childhood. This could lead to isolation and doubt in themselves. Communicating their experience with their children can create various difficulties for both parents and children. Feeling more heard and validated in their experience, feeling that they have the right to be exhausted would allow exhausted parents to seek help earlier, reduce their feeling of guilt and thus act on the impact of the loss of control and on communication in the parent–child relationship. Knowing that the family is an important determinant of health, beyond this individual approach to supporting burnt-out parents, we suggest a more global public health approach, from the angle of health promotion, through protective measures (policies, environment, reorientation of services) that would complement actions on the individual (education, participation...).

## Data availability statement

The raw data supporting the conclusions of this article will be made available by the authors, without undue reservation.

## Ethics statement

The studies involving humans were approved by Comité d’éthique hospitalo-facultaire des Cliniques Universitaires Saint-Luc, UCLouvain. The studies were conducted in accordance with the local legislation and institutional requirements. The participants provided their written informed consent to participate in this study.

## Author contributions

A-CD: Writing – original draft. MR-L: Writing – review & editing. ZM: Writing – review & editing. AF: Writing – review & editing. ML: Writing – review & editing. JM: Writing – review & editing. IA: Writing – review & editing.
